# Multi-omics integration identifies key biomarkers in retinopathy of prematurity through 16S rRNA sequencing and metabolomics

**DOI:** 10.3389/fmicb.2025.1601292

**Published:** 2025-06-18

**Authors:** Linlin Guo, Ruoming Wang, Liping Han, Yongcheng Fu, Xiujuan Wang, Lintao Nie, Wenjun Fu, Hongyan Ren, Lijia Wu, Guangshuai Li, Juan Ding

**Affiliations:** ^1^The Second Department of Radiotherapy, The First Affiliated Hospital of Zhengzhou University, Zhengzhou, China; ^2^Department of Nursing, The First Affiliated Hospital of Zhengzhou University, Zhengzhou, China; ^3^Department of Gynecology, The First Affiliated Hospital of Zhengzhou University, Zhengzhou, China; ^4^Department of Pediatric Surgery, The First Affiliated Hospital of Zhengzhou University, Zhengzhou, China; ^5^Department of Obstetrics, The First Affiliated Hospital of Zhengzhou University, Zhengzhou, China; ^6^Shanghai Mobio Biomedical Technology Co., Ltd., Shanghai, China; ^7^Department of Neonatal Intensive Care Unit, The First Affiliated Hospital of Zhengzhou University, Zhengzhou, China; ^8^Department of Plastic and Reconstructive Surgery, The First Affiliated Hospital of Zhengzhou University, Zhengzhou, China

**Keywords:** retinopathy of prematurity, differential intestinal floral, differentially accumulated metabolites, multi-omics integration, biomarkers

## Abstract

**Background:**

The gut microbiome is increasingly recognized for its role in the pathogenesis of neonatal conditions commonly associated with retinopathy of prematurity (ROP). This study aimed to identify key intestinal microbiota and metabolites in ROP and examine their relationships.

**Methods:**

Fecal samples were collected from infants with and without ROP at weeks 2 (T1) and 4 (T2) for 16S rRNA sequencing. At T2, additional fecal samples underwent non-targeted metabolomic analyses. A combined analysis of the 16S rRNA sequencing and metabolomics data was performed.

**Results:**

No significant differences in α-diversity indexes were observed between the ROP and non-ROP at T1. However, at T2, the Chao, ACE, and Shannon indices were significantly higher, whereas the Simpson index was lower in ROP compared to non-ROP. At the phylum level, the dominant phyla at T2 included *Pseudomonadota*, *Bacillota*, *Actinomycetota*, *Bacteroidota*, and *Verrucomicrobiota*. LEfSe analysis of T2 showed that *Bifidobacterium*, *Rhodococcus*, *Staphyloococcus*, *Caulobacter*, *Sphingomonas*, *Aquabacterium*, and *Klebsiella* as key genera associated with ROP. Metabolomic analysis identified 382 differentially accumulated metabolites, which were enriched in steroid hormone biosynthesis; the PPAR signaling pathway; linoleic acid metabolism; histidine metabolism; and alanine, aspartate, and glutamate metabolism. Additionally, the AUC of the combined analysis exceeded that of differential bacterial communities (0.9958) alone.

**Conclusion:**

This study revealed characteristic changes in the intestinal flora and metabolites in ROP, which provide promising targets/pathways for ROP diagnosis and therapy.

## 1 Introduction

Retinopathy of prematurity (ROP) is a severe disorder characterized by abnormal retinal vascularization, predominantly affecting premature infants, especially those born at extremely low gestational ages or with low birth weights (Blencowe et al., 2013; Arya et al., 2025). ROP is a leading cause of preventable blindness worldwide (Sabri et al., 2022). Additionally, it has led to complications, including myopia, strabismus, glaucoma, and amblyopia (Sabri et al., 2022; Nair et al., 2022). Despite significant advancements in neonatal intensive care medicine, the incidence of ROP remains high, particularly in low- and middle-income countries. In China, the incidence has reached 17.8%, placing a significant economic burden on both families and society (Sabri et al., 2022; Sen et al., 2020). Therefore, understanding these molecular mechanisms is crucial for early diagnosis and treatment.

ROP is a multifactorial disease involving complex interactions between genetic predisposition and environmental factors (Bujoreanu Bezman et al., 2023; Fevereiro-Martins et al., 2022; Ortega-Molina et al., 2015; Shastry, 2010). Emerging evidence suggests that the gut microbiome and metabolic dysregulation also plays a role in its pathogenesis (Zhang et al., 2023; Chang et al., 2025). The gut microbiota, a complex and diverse microbial community residing in the human gastrointestinal tract, is essential for maintaining human health and has been linked to various diseases. The gut microbiome has been widely implicated in the pathogenesis of neonatal conditions that are common among patients with ROP, such as bronchopulmonary dysplasia and necrotizing enterocolitis (Neu and Pammi, 2018; Karaca and Takci, 2025; Sha et al., 2024; Zhang et al., 2022). Moreover, the gut microbiome can crosstalk with distal organs, such as the eye. Recent studies have confirmed an association between the gut microbiome and eye-related diseases via the gut-retina axis in age-related macular degeneration, diabetic retinopathy, and retinitis pigmentosa (Huang et al., 2021; Kutsyr et al., 2021; Rowan and Taylor, 2018). In light of the identification of this axis, microbiome shifts and metabolic changes may be regarded as potential biomarkers and therapeutic targets of ROP (Tomita et al., 2021; Zhou et al., 2020). Chang et al. demonstrated that reduced gut microbial diversity may contribute to the development of ROP in preterm infants (Chang et al., 2025). The α-diversity of fetal samples from infants with ROP is significantly reduced (Westaway et al., 2022). Other studies have suggested that amino acid synthesis is enhanced in the non-ROP group, while Enterobacteriaceae species are more abundant in infants with ROP, with these microbial changes being closely associated with metabolic alterations. Yang et al. identified increased levels of glycine and malonylcarnitine in the blood as promising biomarkers for ROP prediction using targeted blood metabolomics (Yang et al., 2020). Additionally, elevated plasma citrulline levels, aminoadipate, and arginine, along with reduced creatine levels, have been observed in ROP patients (Zhou et al., 2021b). In a prospective study, Nilsson et al. concluded that low concentrations of sphingosine-q-phosphate signaling lipids were associated with a series of ROP (Nilsson et al., 2021). However, changes in the intestinal flora and metabolism of ROP remain largely unknown.

Integrating multi-omics data, such as 16S rRNA sequencing and metabolomics, provides a more comprehensive understanding of the complex biological processes underlying ROP (Chen et al., 2025). In this study, we aimed to characterize the neonatal gut microbiota and metabolic profiles of infants with ROP, and identify biomarkers for its early diagnosis and prevention. By conducting an integrated analysis of 16S rRNA sequencing and metabolomics data, we provide insights into the role of gut microbiota and metabolites in ROP progression, which aid in improving its clinical management.

## 2 Materials and methods

### 2.1 Patients and sample collection

Between March 2023 and June 2024, we conducted a follow-up study on preterm infants born in the Neonatal Department of the First Affiliated Hospital of Zhengzhou University (Zhengzhou, China). Fecal samples from a total of 59 preterm infants were firstly collected at 14 ± 1 days after birth (i.e., at 2 weeks, T1); and then again at 28 ± 1 days after birth (i.e., at 4 weeks, T2). Finally, according to the International Classification of Retinopathy of Prematurity protocol (International Committee for the Classification of Retinopathy of Prematurity, 2005), the preterm infants were divided into ROP (*n* = 29) and non-ROP (*n* = 30) groups. At 2 weeks after birth (T1), 13 and 18 patients were in the ROP and non-ROP groups, respectively. At 4 weeks after birth (T2), 20 and 24 cases were defined as ROP and non-ROP, respectively. Among these, 16 infants (4 ROP and 12 non-ROP) provided fecal samples at both T1 and T2, while the remaining samples were from independent participants. Preterm infants in the non-ROP group remained in the non-ROP state during hospitalization, and all samples were harvested at a single time point without duplication. The research protocols were approved by the Ethics Committee of the First Affiliated Hospital of Zhengzhou University (approval no. 2023-KY-0505-002) and complied with the principles of the Declaration of Helsinki. Informed consent was obtained from the guardians of all participants.

### 2.2 DNA extraction, PCR amplification, and 16S rRNA sequencing

All fecal samples collected at T1 and T2 were subjected to Shanghai Mobio Biomedical Technology Co., Ltd. (Shanghai, China) for 16S rRNA sequencing using the Illumina NextSeq 2000 platform. All samples were processed in a single experimental batch under identical conditions. Briefly, the samples were suspended in 790 μL aseptic lysis buffer (4M guanidine thiocyanate, 250 μL; 10% N-lauroyl sarcosine, 40 μL; 5% N-lauroyl sarcosine-0.1 M phosphate buffer [pH 8.0], 500 μL), and after violent vortex, the samples were incubated at 70°C for 1 h. After incubation, the samples were added with glass beads (0.1 mm, 500–750 μL), mixed, and beaten for 10 min with 25 HZ/s to fully split the cell membrane and nuclear membrane. Then, the E.Z.N.A.^®^ Stool DNA Kit (Omega Bio-Tek, Inc., GA, United States) was used to extract genomic DNA according to the kit instructions, and the isolated DNA concentrations were measured by QUBIT 3.0 (Invitrogen/Q33216, United States). The DNA solution was stored at –20°C for further analysis.

Using the extracted DNA from each sample as a template, the V3-V4 region of 16S rRNA gene was amplified with the primers (341F: 5′-CCTACGGGNGGCWGCAG –3′ and 805R: 5′-GACTACHVGGGTATCTAATCC-3′), PCR amplification was performed in the EasyCycler 96 PCR system (Analytik Jena Corp., AG, Germany). Finally, the amplified products from different samples were pooled in equal proportions and sequenced using the Illumina NextSeq 2000 platform (Illumina, United States).

### 2.3 Analysis of the obtained 16S rRNA sequencing data

The original 16S rRNA sequencing data underwent quality control and denoising using the Divisive Amplitude Denoising Algorithm 2 (DADA2), yielding amplicon sequence variations (ASVs). Based on the SILVA reference database (SSU138.2), the representative sequences of each ASV were annotated and taxonomic analysis was performed to obtain the bacterial composition information of the samples. Alpha diversity was assessed the richness and diversity of the intestinal flora, and diversity indices included the ACE, Chao, Shannon, and Simpson indices. Principal coordinate analysis (PCoA) based on the Bray–Curtis distance was used to compare the differences in community structure between groups. Permutational Multivariate Analysis of Variance (PerMANOVA, also known as Adonis analysis) was used to test the significance of the differences in the overall microbiota between the groups. R software was used to draw bar charts of the bacterial composition in each group at the phylum and genus levels. Linear discriminant analysis Effect Size (LEfSe) (lefse 1.1)^1^ was used to identify different bacterial genera between groups. LEfSe analysis first performs a non-parametric Kruskal–Wallis rank-sum test to detect features with significant differential abundance, followed by linear discriminant analysis (LDA) to estimate the effect size of each feature. The metabolic pathways of the microbial community were predicted using PICRUSt2 analysis.^2^ LEfSe was again used to identify metabolic pathways with significant intergroup differences. Differential pathways were defined as those with a Kruskal–Wallis *p*-value < 0.05 and an LDA score > 3.0. Only pathways meeting both criteria were considered significantly enriched and included in the results. These pathways were ranked in descending order of LDA score to reflect their relative effect sizes.

### 2.4 Isolation of metabolites, and liquid chromatography-mass spectrometry

Based on the 16S rRNA sequencing results, fecal samples at T2 were used for metabolomic analysis. The fecal samples were transferred to 2 mL centrifuge tube, as well as added with 600 μL methanol containing 2-chloro-L-phenylalanine (4 ppm). After vortexing for 30 s, steel balls were added, and the samples were ground in a tissue grinder for 120 s at 50 Hz. After ultrasound for 10 min at room temperature, the samples were centrifuged at 12,000 rpm for 10 min at 4°C. The supernatant was filtered by a 0.22 μm membrane, and transferred into the detection bottle for LC-MS detection.

The LC-MS was performed using a Vanquish UHPLC system (Thermo Fisher Scientific, United States) equipped with an ACQUITY UPLC HSS T3 column (2.1 × 100 mm, 1.8 μm, Waters, Milford, United States), and a high-resolution tandem mass spectrometer Q Exactive Focus (Thermo Fisher Scientific) with electrospray ionization (ESI) ion source. The flow rate was 0.3 mL/min, the injection volume was 2 μL, as well as the column temperature was set at 40°C. In the positive mode, the mobile phases were 0.1% formic acid in acetonitrile (B2) and 0.1% formic acid in water (A2). The gradient elution conditions for the positive mode were 0–1 min, 10% B2; 1–5 min, 10–98% B2; 5–6.5 min, 98% B2; 6.5–6.6 min, 98–10% B2; 6.6–8 min, 10% B2. In the negative mode, the mobile phases were acetonitrile (B3) and 5 mM ammonium formate water (A3). The gradient elution conditions for the negative mode were 0–1 min, 10% B3; 1–5 min, 10–98% B3; 5–6.5 min, 98% B3; 6.5–6.6 min, 98–10% B3; 6.6–8 min, 10% B3. After that, the Thermo Q Exactiv Focus was operated in both positive and negative ion modes. The positive and negative ion spray voltage were respectively 3.50 and –2.5 kV, and the sheath and auxiliary gases were 40 and 10 arb, respectively. The capillary temperature was 325°C, and the first-level full scanning was performed at a resolution of 70,000 with the 100–1,000 m/z ion scanning range. Second-level cracking was performed using the HCD, and the collision energy was 30 eV, with a second-level resolution of 17,500. The top three ions were collected, and unnecessary MS/MS information was removed by dynamic exclusion.

### 2.5 Metabolomics analysis

The raw data generated by LC-MS were converted to mzXML formation using the MSConvert tool in Proteowizard package (v.3.0.8789), and the XCMS software in R was used for peak detection, peak filtering, and peak alignment to obtain a quantitative list of substances, with the parameters of bw = 2, ppm = 15, peakwidth = c (5, 30), mzwid = 0.015, mzdiff = 0.01, as well as method = “centWave.” Random forest correction based on quality control (QC) samples was used to eliminate systematic errors, and substances with a relative standard deviation (RSD) of less than 30% in the QC samples were retained for subsequent analysis. Principal component analysis (PCA) was then performed for outlier detection and batch effect evaluation using the preprocessed dataset. Orthogonal partial least squares discriminant analysis (OPLS-DA) was conducted to discriminate variables between the groups. Afterward, significantly differentially accumulated metabolites (DAMs) were identified based on the thresholds of VIP > 1 and *P* < 0.05, and then subjected to Kyoto Encyclopedia of Genes and Genomes (KEGG) pathway enrichment analysis with *P* < 0.05, which was considered statistically significant.

### 2.6 Conjoint analysis of 16S rRNA sequencing data and metabolomics data

The Spearman correlation between the differential bacterial communities and the top 20 DAMs with the highest VIP values (identified via non-targeted metabolomics) was calculated using the rcorr function of the Hmisc package. The correlation heatmap was generated using the heatmap.2 function from the gplots package. The lm function of the stats package was used to calculate the fitting linear model of the differential bacterial communities and DAMs, and the ggplot function of the ggplot2 package was used to plot the correlation between the two. Redundancy analysis (RDA) was performed using the decorana function in the vegan package (default parameter eigenvalue decomposition method), and the ggplot function in the ggplot2 package was used to draw RDA plots of microbiota, metabolites, and samples. Finally, a random forest model was constructed using the ranger function in the ranger package, and the ggplot function in the ggplot2 package was used to draw the receiver operating characteristic (ROC) curve.

### 2.7 Statistical analysis

The data were reported as mean ± SD unless specified. Chi-square or Fisher’s exact test for categorical variables, and Student’s *t*-test or Mann–Whitney U test for continuous variables depending on data distribution. No multiple testing correction was applied. *P* < 0.05 was deemed to be statistically significant. Statistical analysis was conducted using GraphPad Prism 9 (GraphPad Software, United States).

## 3 Results

### 3.1 Clinical information of the included subjects

The clinical characteristics of the included subjects are summarized in Table 1. The gestational age/hospital stay of ROP and non-ROP patients were 29.55 ± 1.75 weeks/56 days, and 31.83 ± 1.52 weeks/34 days, respectively. These results indicated that the gestational age of the ROP patients was significantly lower and the hospital stay was longer (*P* < 0.05) than that of the non-ROP patients. Additionally, the birth weight of ROP infants was significantly lower (*P* < 0.05) than that of non-ROP infants, whereas the fraction of inspiration O_2_ (FiO_2_) in ROP infants was markedly higher than that in non-ROP infants (*P* < 0.05) (Table 1). Furthermore, 29.41% of the ROP patients used probiotics after birth, and none of the non-ROP patients used probiotics (Table 1). To minimize the variability introduced by different antibiotic types, all enrolled cases received the same class of antibiotics (cephalosporins).

**TABLE 1:** The clinical characteristics of the included subjects.

	ROP	Non-ROP	*P*
Gestational age (mean ± SD, weeks)	29.55 ± 1.75	31.83 ± 1.52	0.0005
Birth weight (g)	1162.94 ± 286.22	1680.66 ± 443.08	0.0004
Gender (male, n%)	(16/29) 55.17%	(24/30) 80%	0.0781
Hospital stay (day)	56	34	0.0004
Delivery mode (cesarean, n%)	(24/29) 82.75%	(30/30) 100%	0.0237
Feeding strategy (breast milk, n%)	(14/29) 48.27%	(24/30) 80%	0.0231
Ventilation mode (single, n%)	(10/29) 34.48%	(14/30) 46.67%	0.4919
Fraction of inspiration O_2_ (FiO_2_)	31.29	23.8	0.0058
Red blood cell (First time)	3.74 × 10^12^/L	3.73 × 10^12^/L	0.9528
Blood platelet (First time)	232 × 10^9^/L	223 × 10^9^/L	0.7968
CRP (First time)	0.99 mg/L	2.06 mg/L	0.237
Transfusion (Yes, n%)	(22/29) 75.86%	(16/30) 53.33%	0.1248
First delivery (Yes, n%)	(14/29) 48.27%	(14/30) 46.67%	1
Pulmonary dysplasia	(27/29) 93.10%	(30/30) 100%	0.2373
Use probiotics (n%)	(11/29) 37.93%	(0/30) 0	0.0007
Use antibiotics* (n%)	(29/29) 100%	(30/30) 100%	/

*All enrolled cases received the same class of antibiotics (cephalosporins).

Representative fundus photographs of ROP patients and controls can be found in Supplementary Figure 1. In our cohort, the majority of infants diagnosed with ROP presented with relatively mild disease (Table 2). Specifically, 68% of the cases were classified as stage 1, while the remainder were stage 2. Most cases were located in Zone II, with a smaller proportion in Zone III. Additionally, the presence of plus disease was uncommon, observed in only 13% of ROP infants. Overall, the ROP group in our study primarily consisted of early-stage, mid-peripheral cases without significant vascular dilation or tortuosity.

**TABLE 2:** The clinical characteristics of enrolled ROP cases.

Clinical indicators	Classification	Percentage
Stage	1	(20/29) 68.96%
	2	(9/29) 31.04%
Zone	II	(22/29) 75.86%
	III	(7/29) 24.14%
Plus disease	–	(25/29) 86.21%
	+	(4/29) 13.79%

### 3.2 The overall structure of intestinal flora in ROP samples at T1 and T2 times

**FIGURE 1:**
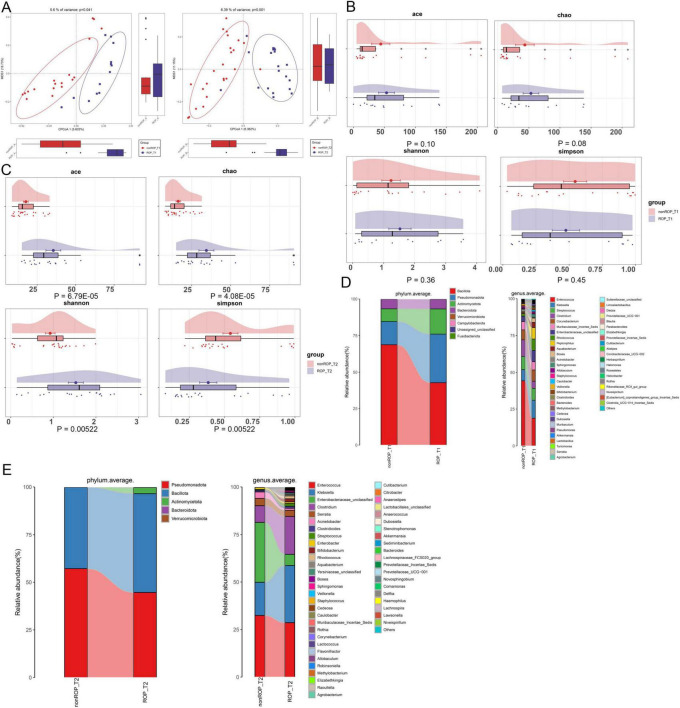
The overall structure of intestinal flora in retinopathy of prematurity (ROP) samples at T1 and T2 times. **(A)** Principal coordinate analysis (PCoA) based on the Bray–Curtis distance to compare the differences in community structure between the two groups at T1 and T2. **(B)** The α-diversity including ACE, Chao, Shannon, and Simpson indexes in the two different groups at the T1 time. **(C)** The α-diversity including ACE, Chao, Shannon, and Simpson indexes in the two different groups at the T2 time. **(D)** Dominant phyla and top50 genera in the different groups at T1. **(E)** Dominant phyla and top50 genera in the different groups at T2.

To explore the role of the intestinal flora in patients with ROP, fecal samples from patients with ROP and non-ROP individuals at T1 and T2 were collected for 16S rRNA sequencing. As shown in Figure 1A, the PCoA results revealed that there were significant differences in clustering between the ROP and non-ROP samples, regardless of T1 or T2 (*P* = 0.041 in T1 and *P* = 0.001 in T2). These results suggest that these samples are suitable for further sequencing and analysis. After annotation, there were 315 and 287 ASVs in the non-ROP and ROP samples, respectively, at T1, with 244 overlapping ASVs, and 83 and 195 ASVs in the non-ROP and ROP samples, respectively, with 77 shared ASVs at T2. Thereafter, the indexes of α-diversity at the T1 and T2 times were analyzed, containing Chao, Shannon, ACE, as well as Simpson indexes. At T1, no significant differences were found in the ACE, Chao, Shannon, and Simpson indices between the ROP and non-ROP samples (*P* > 0.05) (Figure 1B). At T2, compared with the non-ROP samples, the ACE Chao, and Shannon indices were significantly increased (*P* < 0.05), whereas the Simpson index was significantly reduced in the ROP samples (*P* < 0.05) (Figure 1C). This indicates that the diversity of intestinal flora between the ROP and non-ROP groups was significantly different only at week 4 (T2), which is consistent with the fact that ROP is generally diagnosed at week 4. Additionally, it can be inferred that at week 4, the α-diversity of intestinal flora could be enhanced in ROP infants compared with the non-ROP individuals.

### 3.3 The specific composition of intestinal flora at phylum and genus levels in ROP samples at T1 and T2

Furthermore, the specific composition of the intestinal flora was investigated at the phylum and genus levels at both T1 and T2. At T1, annotated ASVs belonged to the phyla *Bacillota*, *Pseudomonadota*, *Actinomycetota*, *Bacteroidota*, *Verrucomicrobiota*, and *Campylobacterota* (Figure 1D). The top50 genera at T1 are shown in Figure 1D and include *Enterococcus*, *Klebsiella*, *Streptococcus*, *Clostridium*, *Bifidobacterium*, *Sphingomonas*, *Staphyloococcus*, *Akkermansia*, *Lactobacillus*, and *Alistipes*. However, in T2, the annotated ASVs were assigned to five dominant phyla: *Pseudomonadota*, *Bacillota*, *Actinomycetota*, *Bacteroidota*, and *Verrucomicrobiota* (Figure 1E). The top50 genera were *Enterococcus*, *Klebsiella*, *Clostridium*, *Serratia*, *Acinetobacter*, *Clostridioides*, *Streptococcus*, *Bifidobacterium*, *Rhodococcus*, *Staphyloococcus*, *Lactococcus*, *Akkermansia*, *Flavonofractor*, and *Lachnospira* (Figure 1E).

**FIGURE 2:**
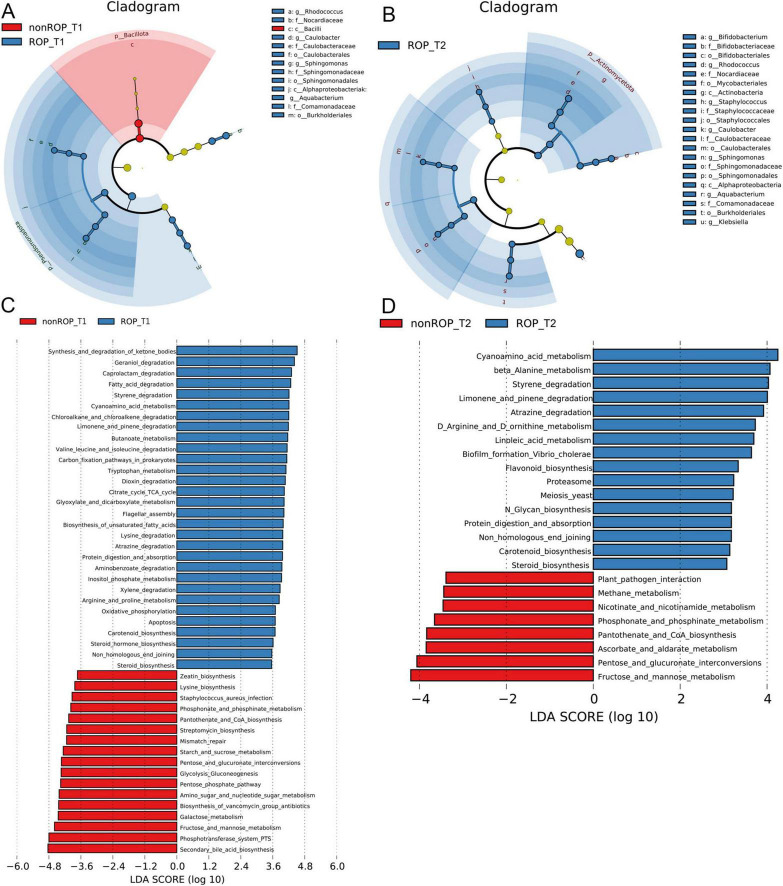
The specific composition and functions of intestinal flora at phylum and genus levels in ROP samples at T1 and T2 times. **(A)** Crucial bacterial communities in the ROP at T1 using LEfSe. **(B)** Crucial bacterial communities in the ROP at T2 using LEfSe. **(C)** Functional analysis of the identified bacterial communities at T1 as predicted by PICRUSt2 analysis. **(D)** Functional analysis of the identified bacterial communities at T2 as predicted by PICRUSt2 analysis.

Subsequently, LEfSe was conducted to analyze the crucial bacterial communities in the ROP at T1 and T2. At T1, the crucial genera in the ROP group were *Rhodococcus*, *Caulobater*, *Sphingomonas*, and *Aquabacterium*, whereas the key bacterial community in the non-ROP group was *Bacilli* (Figure 2A). In addition, at T2, *Bifidobacterium*, *Rhodococcus*, *Staphyloococcus*, *Caulobacter*, *Sphingomonas*, *Aquabacterium*, and *Klebsiella* were the pivotal genera for ROP (Figure 2B).

### 3.4 Functional analysis of the identified intestinal flora in ROP

**FIGURE 3:**
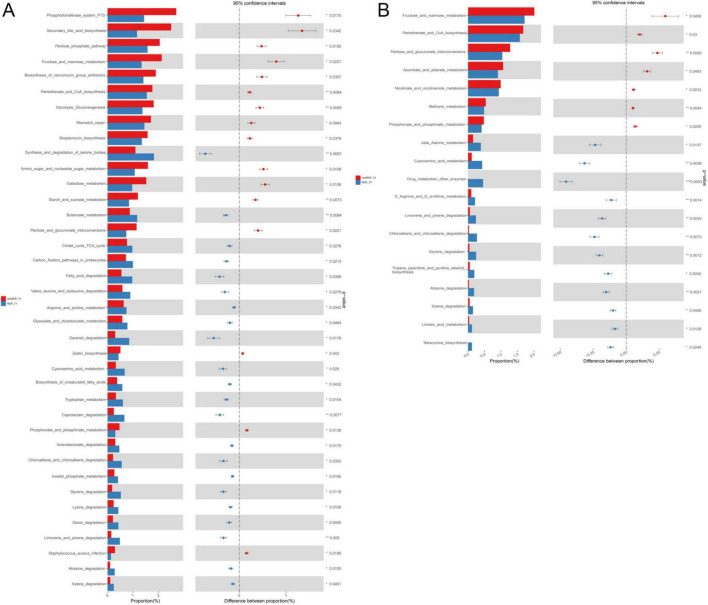
The statistical analysis of the pathways enriched by the identified bacterial communities. **(A)** Significant differences in the metabolic pathways enriched by the bacterial communities identified at T1 by LEfSe analysis. **(B)** Significant differences in the metabolic pathways enriched by the bacterial communities identified at T2 by LEfSe analysis.

The functions of the intestinal flora identified by 16S rRNA sequencing were further analyzed. At T1, the identified intestinal flora in the ROP group were significantly enriched in “fatty acid degradation,” “chloroalkane and chloroalkene degradation,” “butanoate metabolism,” “valine leucine and isoleucine degradation,” “citrate cycle (TCA cycle),” “carbon fixation pathway in prokaryotes,” “oxidative phosphorylation,” “apoptosis,” “arginine and proline metabolism,” “carotenoid biosynthesis,” and “steroid hormone biosynthesis”; as well as the identified intestinal flora in non-ROP group were evidently enriched in “zeatin biosynthesis,” “lysine biosynthesis,” “phosphonate and phosphinate metabolism,” “mismatch repair,” “pentose phosphate pathway,” and “secondary bile acid biosynthesis” (Figure 2C). At the T2 time, the identified intestinal flora in ROP group were closely associated with “cyanoamino acid metabolism,” “limonene and pinene degradation,” “carotenoid biosynthesis,” “linoleic acid metabolism,” “flavonoid biosynthesis,” as well as “D-arginine and D-ornithine metabolism.” Furthermore, the identified intestinal flora in non-ROP group were mainly related to “pantothenate and CoA biosynthesis,” “plant pathogen interaction,” “methane metabolism,” as well as “fructose and mannose metabolism” (Figure 2D). Statistical analyses of the predicted pathways at T1 and T2 are shown in Figures 3a,b. At the T2 time, we observed that there were 19 significantly enriched pathways between the ROP and non-ROP groups (*P* < 0.05), including 7 activated pathways in non-ROP group (such as “fructose and manse metabolism,” “pentose and glucuronate interconversions,” as well as “pantothenate and CoA biosynthesis”), and 12 activated pathways in ROP group (such as “cyanoamino acid metabolism,” “limonene and pinene degradation,” “linoleic acid metabolism,” “tetracycline biosynthesis,” as well as “D-arginine and D-ornithine metabolism”) (Figure 3B).

### 3.5 Sub-analysis of 16S rRNA sequencing within the ROP group at T2

To further assess the potential impact of probiotic administration on the gut microbiota, we performed a sub-analysis of 16S rRNA sequencing within the ROP group at time point T2, comparing infants who received probiotics versus those who did not. The probiotic (PE_ROP) and non-probiotic (NP_ROP) subgroups showed partial separation on PCoA plots (Supplementary Figure 2A), suggesting potential shifts in overall microbial community structure. However, there was notable overlap between the groups, indicating that the differences were not pronounced. Alpha-diversity analysis revealed that while no statistically significant differences were found between the two subgroups on the Chao, Shannon, ACE, Simpson indices, a decreasing trend in alpha-diversity was observed in the probiotic group (Supplementary Figure 2B). Notably, this trend moved in the direction of the non-ROP group, which had significantly lower alpha-diversity than the ROP group in the main analysis. This suggests that probiotic use may have shifted the ROP microbiota toward a more non-ROP-like profile. Further dissecting the genus composition of the intestinal flora, we observed reduced relative abundance of *Klebsiella* and *Enterococcus* (commonly considered opportunistic pathogens) in the probiotic group, alongside increased levels of *Bifidobacterium* and *Clostridium*, which are associated with intestinal health (Supplementary Figures 2C,D). However, these differences did not reach statistical significance.

### 3.6 Identification and functional analysis of DAMs in ROP samples at T2 time

**FIGURE 4:**
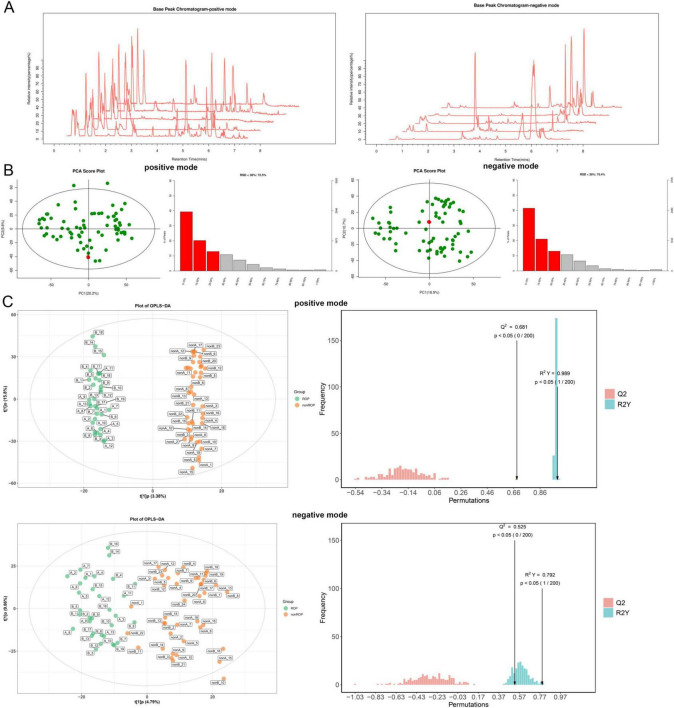
Quality control and quality assessment of all the samples at the T2 time. **(A)** Base peak chromatograms for positive and negative modes. **(B)** Quality control and proportions of characteristic peaks with a relative standard deviation (RSD) < 30% in the positive and negative modes of all samples using Principal Component Analysis. **(C)** The score diagram and displacement test diagram of orthogonal partial least squares discriminant analysis (OPLS-DA) based on fecal samples from T2 blood serum samples in positive and negative modes.

Because of the significant diversity of the intestinal flora at week 4 (T2), we further performed metabolomic analysis of the fecal samples at T2. Base peak chromatograms for the positive and negative modes were displayed in Figure 4A. The PCA results showed that the QC samples were densely distributed and reproducible in both positive and negative modes (Figure 4B), indicating that the system was stable and the data were reliable. In QC samples, the proportions of characteristic peaks with RSD < 30% under positive and negative conditions were 72.5 and 75.4%, respectively (Figure 4B), illustrating that the metabolomics data were reliable and conducive to the detection of biomarkers. In addition, the OPLS-DA models in the positive and negative conditions showed that the samples in the ROP and non-ROP groups could be significantly differentiated, and the values of R2/Q2 in the positive and negative modes were 0.989/0.681 and 0.792/0.525, respectively (Figure 4C), indicating that the evaluation model was effective and reliable and could be applied for subsequent secondary structure analysis.

**FIGURE 5:**
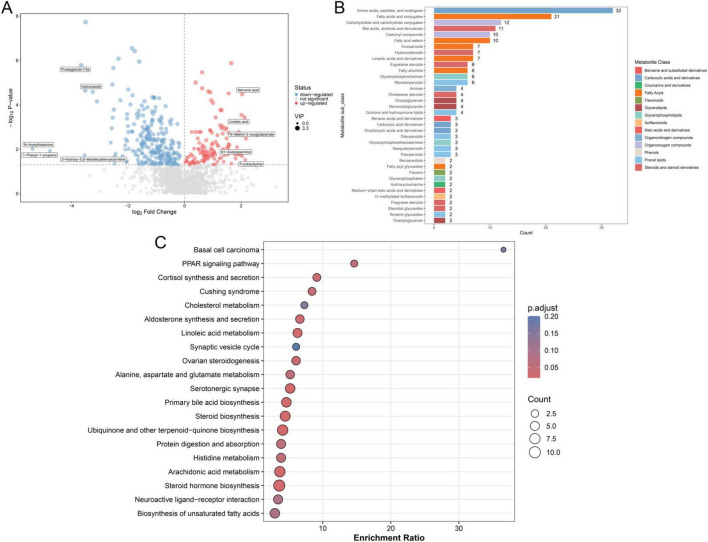
Identification and functional analysis of the significantly differential accumulated metabolites (DAMs). **(A)** Volcano plot of identified DAMs based on thresholds of VIP > 1 and *P* < 0.05. **(B)** Classification of identified DAMs. **(C)** Kyoto Encyclopedia of Genes and Genomes (KEGG) pathway enrichment analysis of the identified DAMs with *P* < 0.05, which was considered statistically significant.

Based on the screening criteria, 382 DAMs were filtered between the ROP and non-ROP groups, including 119 upregulated (nervonic acid, linoleic acid, N-methyl-2-oxoglutaramate, N1-acetylspermine, and fructosyllysine) and 263 downregulated (prostaglandin F3a, iodiconazole, N-acetylhistamine, 1-phenyl-1-propanol, and 3-hydroxy-5,8-tetradecadienoylcarnitine) DAMs in ROP patients (Figure 5A). The screened DAMs were classified into different categories, including amino acids, peptides and analogs, fatty acids and conjugates, carbohydrates and carbohydrate conjugates, bile acids, alcohols and derivatives, carbonyl compounds, and fatty acid esters (Figure 5B). Furthermore, these identified DAMs were subjected to KEGG pathways, and were found to be closely related to “PPAR signaling pathway,” “cortisol synthesis and secretion,” “linoleic acid metabolism,” “protein digestion and absorption,” “steroid hormone biosynthesis,” “histidine metabolism,” as well as “alanine, aspartate and glutamate metabolism” (Figure 5C).

### 3.7 Conjoint analysis of identified differential bacterial communities and DAMs

**FIGURE 6:**
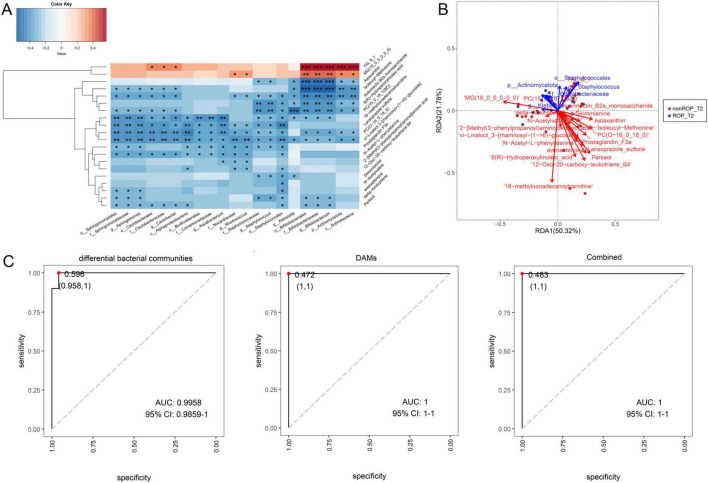
Conjoint analysis of the identified differential bacterial communities by LEfSe analysis, and the top 20 DAMs with the highest VIP values by non-target metabolomics. **(A)** Spearman’s correlation heatmap of the differential bacterial communities and DAMs. **(B)** Redundancy analysis (RDA) of microbiota, metabolites, and samples. The acute angle between the two variables represents a positive correlation; that is, a synergistic effect. The obtuse angle between the two variables indicated a negative correlation; that is, an antagonistic effect. **(C)** Receiver operating characteristic (ROC) curves and area under the curve (AUC) values of the differential bacterial communities identified by LEfSe analysis, top 20 DAMs, and combinations.

Furthermore, to unearth the interactions between the differential bacterial communities in T2 and the top20 DAMs, a conjoint analysis of 16s rRNA sequencing and metabolomics data was carried out. The Spearman correlation analysis observed *Sphingomonas* was significantly negatively correlated to isoleucyl-methionine, avermectin B2a monosaccharide, xi-Linalool 3-[rhamnosyl-(1- > 6)-glucoside], PC(15_0_18_1(9Z)), avenastenone, delta-amorphene, parkeol, 18-methylnonadecanoylcarnitine, lansoprazole sulfone, PC(O-16_0_18_0), prostaglandin F3a, and N-acetyl-L-phenylalanine (*P* < 0.05) (Figure 6A). The genus of *Caulobacter* had significantly negative correlation with parkeol, N-acetyl-L-phenylalanine, xi-Linalool 3-[rhamnosyl-(1- > 6)-glucoside], lansoprazole sulfone, PC(O-16_0_18_0), isoleucyl-methionine, avermectin B2a monosaccharide, PC(15_0_18_1(9Z)), 18-methylnonadecanoylcarnitine, and lansoprazole sulfone; while had significantly positive correlation with FAL8_1 (*P* < 0.05) (Figure 6A). The genera *Aquabacterium* and *Rhodococcus* were significantly negatively correlated with lansoprazole sulfone, PC(O-16_0_18_0), xi-linalool 3-[rhamnosyl-(1- > 6)-glucoside], prostaglandin F3a, N-acetyl-L-phenylalanine, and 2-[methyl(3-phenylpropanoyl)amino]benzoic acid (*P* < 0.05) (Figure 6A). The genus of *Staphyloococcus* had significantly negative correlation with astaxanthin, avermectin B2a monosaccharide, xi-Linalool 3-[rhamnosyl-(1- > 6)-glucoside], 8(R)-hydroperoxylinoleic acid, PC(15_0_18_1(9Z)), 18-methylnonadecanoylcarnitine, lansoprazole sulfone, PC(O-16_0_18_0), prostaglandin F3a, skimmianine, and delta-amorphene (*P* < 0.05) (Figure 6A). *Klebsiella* was significantly negatively correlated with astaxanthin, isoleucyl-methionine, avermectin B2a monosaccharide, 8(R)-hydroperoxylinoleic acid, PC(15_0_18_1(9Z)), 18-methylnonadecanoylcarnitine, xi-linalool 3-[rhamnosyl-(1- > 6)-glucoside], and skimmianine levels (*P* < 0.05) (Figure 6A). For *Bifidobacterium*, it was significantly positively correlated with FAL8_1 and MG(18_0_0_0_0_0); whereas was evidently negatively correlated with xi-Linalool 3-[rhamnosyl-(1- > 6)-glucoside], astaxanthin, avermectin B2a monosaccharide, 8(R)-hydroperoxylinoleic acid, PC(15_0_18_1(9Z)), 18-methylnonadecanoylcarnitine, PC(O-16_0_18_0), delta-amorphene, and parkeol (*P* < 0.05) (Figure 6A). RDA analysis showed that *Klebsiella* had synergistic effects with MG(18_0_0_0_0_0), delta-amorphene, and 18-methylnonadecanoylcarnitine; while had antagonistic effects with astaxanthin, isoleucyl-methionine, prostaglandin F3a, lansoprazole sulfone, and parkeol (Figure 6B). Finally, the AUC values of differential bacterial communities, DAMs, as well as combination of differential bacterial communities and DAMs were 0.9958, 1, and 1, respectively (Figure 6C). These findings suggest that the predictive effect of conjoint analysis or DAMs is better than that of differential bacterial communities alone.

## 4 Discussion

In this study, through a comprehensive analysis of multi-omics data integrating 16S rRNA sequencing and metabolomics, we successfully identified key biomarkers of ROP, injecting new impetus into the ROP research field. Historically, ROP has been a challenging topic in neonatology because of its intricate pathogenesis (Gilbert, 2023). Conventional research methods, limited by single-dimensional analysis, have struggled to comprehensively dissect the multifactor interactions involved in ROP progression. The integrated application of multi-omics technologies in this study enabled us to conduct in-depth analyses of ROP pathogenesis from both microbial community structure and metabolite perspective. 16S rRNA sequencing delineates the dynamic characteristics of the gut microbial community, whereas metabolomics precisely captures the metabolite perturbations associated with the development of ROP (Loke et al., 2018). This multi-dimensional and systematic research strategy not only addresses the limitations of previous single-technology studies, but more importantly, establishes for the first time a close link between the intestinal microbial community and metabolite profiles. This provides a new perspective for a deeper understanding for ROP pathogenesis and is expected to advance early diagnosis and precise intervention in clinical practice.

ROP is a destructive neonatal retinal neurovascular disease that leads to visual impairment or even blindness (Sen et al., 2020). Our clinical data also showed that patients with ROP had a lower gestational age, lower birth weight, and longer hospital stay. In China, the screening time for ROP is set at 4–6 weeks after birth (Li et al., 2022), and we found that the diversity of intestinal flora in the ROP group was significantly different from that in the non-ROP group at week 4. Additionally, the current study also showed the α-diversity of intestinal flora in ROP was significantly increased at week 4. A previous study demonstrated that type 1 ROP showed no significant difference in microbial diversity at 8 weeks after birth, whereas type 2 ROP displayed enhanced diversity compared with non-ROP, indicating that increased gut microbial diversity may be linked to the evolution of ROP in high-risk preterm infants (Chang et al., 2025). Subsequently, the transformation in the specific composition of intestinal flora at week 4 in ROP was assessed at both the phylum and genus levels. At the phylum level, Pseudomonadota, Bacillota, Actinomycetota, Bacteroidetes, and Verrucomicrobiota were the five most dominant phyla. At the genus level, *Bifidobacterium*, *Rhodococcus*, *Staphyloococcus*, *Caulobacter*, *Sphingomonas*, *Aquabacterium*, and *Klebsiella* are crucial bacterial communities in ROP.

Among the taxa enriched in the ROP group, particularly *Staphyloococcus*, *Sphingomonas*, and *Klebsiella*, have been implicated in neonatal infections and are known to promote systemic inflammation in premature infants (Thänert et al., 2024; McCartney and Hoyles, 2023; Stewart et al., 2013). Systemic inflammation impairs retinal health, potentially contributing to the development of ROP. Moreover, in neonatal contexts, increased gut permeability may facilitate microbial translocation, further exacerbating inflammatory responses that are known to disturb retinal vascular homeostasis.

*Bifidobacterium*, maintains intestinal barrier function, promotes immune cell development, and regulates cytokine secretion, but also participates in regulating short-chain fatty acid metabolism and improving the oxidative stress response (Laursen et al., 2021). Another study illustrated that *Bifidobacterium bifidum* CCFM1163 could increase the content of short-chain fatty acids (especially propionic acid), the abundance of *Romboutsia*, *Faecalibaculum*, *Turicibacter*, and *Bifidobacterium* in the feces, and upregulate the expression of enteric nerve marker proteins, thereby restoring intestinal barrier function, promoting intestinal motility, and alleviating cathartic colon (Tang et al., 2023). A previous study has found that Bifidobacterium genera was notably less abundant in type 1 ROP group at first postnatal week and remained low in subsequent weeks (Chang et al., 2025). However, in our study Bifidobacterium was elevated in ROP group, which is likely attributed to the fact that a proportion of infants in the ROP group received probiotic supplementation. Our subgroup analysis further confirmed this. *Rhodococcus* strains are widely present in both natural and anthropogenic environments owing to their high metabolic multifunctionality, biodegradation activity, and adaptability to various stress conditions. Moreover, the ability of *Rhodococcus* strains to generate higher value-added products has attracted considerable attention, particularly in relation to lipid accumulation (Donini et al., 2021). Pinna et al. (2021) showed that *Rhodococcus*, *Alistipes*, *Lactobacillus*, and *Alloprevotella* are significantly enriched in individuals with prediabetes. *Staphyloococcus*, pathogens in the gut, can cause intestinal infection, lead to an imbalance in intestinal flora, and participate in the occurrence and progression of inflammatory bowel disease (Buffet-Bataillon et al., 2022). A previous investigation showed that *Staphylococcus aureus* not only has the capacity to produce various enterotoxins and other toxins to activate inflammatory cells and trigger inflammatory responses, but also that activated inflammatory cells can express multiple cytokines and induce inflammatory responses (Chen et al., 2022). A prior research by Zhou et al. (2022) reported the lower abundances of *Caulobacter* as well as *Novosphingobium* in colon cancer tissues had longer survival rates. *Sphingomonas* is a Gram-negative bacterium that can reduce lung inflammation by activating sphingolipids, which are involved in maintaining retinal cell structure and function, regulating angiogenesis, and antioxidant and anti-inflammatory activities (Li et al., 2020). Wu et al. (2023) observed that compared with the healthy children, the α-diversity in children with acute bronchiolitis was lower, as well as the abundance of *Sphingomonas*, a bacterium that produces sphingolipid, was increased in children with acute bronchiolitis. *Aquabacterium*, *Lactobacillus*, and *Prevotella* have been identified as crucial genera in cervical cancer (Xu et al., 2022). It has been reported that overgrowth of *Klebsiella* in the gut is highly predictive of brain damage as well as of pro-inflammatory immune tone (Seki et al., 2021). Together with our findings, these reports suggest that *Bifidobacterium*, *Rhodococcus*, *Staphyloococcus*, *Caulobacter*, *Sphingomonas*, *Aquabacterium*, and *Klebsiella* are potential candidate biomarkers of ROP. However, the mechanisms underlying the screened candidate biomarkers require further investigation.

Metabolites from the gut microbiome are pivotal hubs that are linked to intestinal flora and disease progression, primarily by reshaping the tumor microenvironment and regulating key signaling pathways in cancer cells as well as various immune cells (Yang et al., 2023; He et al., 2025). In this study, fecal samples at T2 were also employed for metabolomic analysis, and 119 increased DAMs (nervonic acid, linoleic acid, N-methyl-2-oxoglutaramate, N1-acetylspermine, and fructosyllysine) and 263 decreased DAMs (prostaglandin F3a, iodiconazole, N-acetylhistamine, 1-phenyl-1-propanol, and 3-hydroxy-5,8-tetradecadienoylcarnitine) in ROP were significantly enriched in a number of pathways, such as the PPAR signaling pathway, linoleic acid metabolism.

Specifically, the PPAR signaling pathway comprises multiple isoforms (PPAR-α, PPAR-γ, and PPAR-β/δ) and the role of PPAR signaling in ROP is context- and isoform-dependent. PPAR-γ is generally protective in ROP due to its anti-inflammatory and anti-angiogenic effects. Studies have shown that suppression of PPAR-γ is associated with the development of oxygen-induced retinopathy (OIR), suggesting that its downregulation may contribute to disease progression (Tawfik et al., 2009). Furthermore, activation of PPAR-γ has been reported to inhibit pathological neovascularization in various retinal disease models (Ciudin et al., 2013; Zhang et al., 2015). PPAR-β/δ, on the other hand, may play a more complex or even pro-angiogenic role. PPAR-β/δ regulates angiogenic cell behavior in OIR and may promote preretinal neovascularization through mechanisms involving Angptl4. Pharmacological inhibition of this isoform was proposed as a potential therapeutic strategy (Capozzi et al., 2013). PPAR-α has a less defined role in ROP but is known to regulate lipid metabolism and reduce oxidative stress. Activation of PPAR-α, such as through the use of fenofibrate, was found to reduce retinal and choroidal neovascularization, in part through inhibition of CYP2C activity (Gong et al., 2016).

In our metabolomic analysis, linoleic acid was found to be significantly elevated in the ROP group at T2. Linoleic acid, a polyunsaturated omega-6 fatty acid, has been increasingly recognized as a bioactive lipid involved in the early stages of angiogenesis (Samson et al., 2020). Linoleic acid—along with oleic acid and cholesterol—serves as a natural initiator of angiogenesis by creating a permissive lipid microenvironment. This lipid-mediated priming occurs upstream of classical proangiogenic signals such as VEGF, angiopoietin, and erythropoietin. The accumulation of linoleic acid may therefore contribute to a pro-angiogenic metabolic milieu in premature infants predisposed to ROP, particularly during the avascular-to-neovascular switch phase of the disease. These findings support the hypothesis that metabolic reprogramming, including lipid imbalance, may play an underappreciated role in promoting pathological retinal neovascularization in ROP.

Postpartum systemic steroid hormones can increase the risk of ROP development. The development of severe ROP can be prevented by focusing on the methods of systemic steroid hormone administration and avoiding excessive doses in infants born with PMA < 28 weeks (Tao, 2022). Histidine metabolism is observed to be involved in the pathogenesis of diabetic retinopathy (Becker et al., 2021). Zhou et al. (2021a) found that aspartate, alanine, and glutamate metabolism was closely associated with the progression of oxygen-induced retinopathy by analyzing mouse retinas using non-targeted metabolomics. Additionally, blood metabolomics of diabetic retinopathy suggested that elevated levels of glutamate, n-acetyl-L-glutamate, aspartate, n-acetyl-L-aspartate, and glutamine as well as decreased levels of docosahexaenoic acid, dihomo-gamma-linolenic acid, and eicosapentaenoic acid have been identified as metabolic features that distinguish diabetic retinopathy from non-diabetic retinopathy, whereas phosphatidylcholine and 13-PHODE are the two primary metabolic markers of diabetic retinopathy (Wang et al., 2022). Taken together, we speculate that the increased levels of nervonic acid, linoleic acid, N-methyl-2-oxoglutaramate, N1-acetylspermine, and fructosyllysine, and reduced levels of prostaglandin F3a, iodiconazole, N-acetylhistamine, 1-phenyl-1-propanol, and 3-hydroxy-5,8-tetradecadienoylcarnitine may be closely associated with ROP progression, and the enriched pathways of steroid hormone biosynthesis, PPAR signaling, linoleic acid metabolism, histidine metabolism, and alanine, aspartate, and glutamate metabolism. Nevertheless, the detailed functions of the identified DAMs and the pathways involved in ROP should be further explored.

We further investigated the relationship between the identified intestinal flora at T2 and the screened DAMs. Yang et al. (2022) combined 16S rRNA sequencing and non-targeted metabolomics in the primary Sjogren’s syndrome, as well as observed the abundance of *Escherichia-Shigella* was significantly correlated with high levels of four metabolites (aflatoxin M1, glycocholic acid, L-histidine and phenylglyoxylic acid) through Pearson correlation coefficient analysis. Another study of correlation analysis between the intestinal flora and DMAs demonstrated that in lung cancer, the abundance of *Lachnospira*, *Fusicatenibacter*, and *Firmicutes* was positively correlated with the metabolites of 9,10,13-TriHOME, 3-Oxocholic acid, picolinic acid, and dodecanedioic acid, whereas the metabolites of 9-tridecynoic acid, dodecanedioic acid, 3beta, 12alpha-dihydroxychol-5-en-24-oic acid, and 1,4′-bipiperidine-1′-carboxylic acid were negatively associated with *Ruminococcus gnavus* (Lu et al., 2023). In this study, lansoprazole sulfone, PC(O-16_0_18_0), prostaglandin F3a, N-acetyl-L-phenylalanine, and xi-linalool 3-[rhamnosyl-(1- > 6)-glucoside] negatively correlated with *Sphingomonas*, *Caulobacter*, *Aquabacterium*, *Rhodococcus*, *Klebsiella*, *Bifidobacterium*, and *Staphyloococcus*. However, *Bifidobacterium* had positively correlated with FAL8_1 and MG(18_0_0_0_0_0). Additionally, the AUC value of the combined analysis was higher than that of the differential bacterial community (0.9958). These outcomes imply that the predictive effect of combined analysis may be better for ROP diagnosis and prognosis and that the interaction of intestinal flora and the relevant metabolites may affect the physiological and pathological processes of ROP.

One of the key strengths of our study lies in its integrative multi-omics approach, which combines longitudinal gut microbiota and metabolomic profiling to explore their potential involvement in ROP pathogenesis. By collecting fecal samples at two critical early time points (2 and 4 weeks postnatally), we were able to capture dynamic shifts in microbial and metabolic signatures during a vulnerable developmental window. Furthermore, strict inclusion criteria—such as the exclusion of infants with major comorbidities and consistent use of cephalosporin antibiotics—helped minimize confounding variables. This study provides novel insights into the gut-retina axis in preterm infants and lays important groundwork for future mechanistic and interventional research.

However, several limitations remain. First, while the diagnosis of ROP was made by ophthalmologists based on standardized clinical protocols, our data collection ended before 45 weeks of corrected gestational age, when retinal vascularization is typically complete. The possibility remains that some late-onset ROP cases may have been missed. This potential misclassification bias should be addressed in future studies with extended follow-up periods. Second, this study included a mixture of repeated and independent samples across the two time points, which may introduce certain limitations in interpreting temporal changes, and future studies with fully longitudinal sampling and paired analysis are warranted to better capture individual-level dynamics. Third, all infants were diagnosed with stage 1 or stage 2 ROP; no advanced-stage cases were present. Given the limited number of cases and the absence of higher-stage ROP, subgroup analysis based on ROP stage would lack sufficient statistical power and clinical significance. Future studies with larger and more diverse cohorts are needed to explore microbial or metabolic differences across ROP severity. Fourth, the use of probiotics in a subset of infants within the ROP group might introduce the potential confounding effect. Although our subgroup analysis indicate that the probiotic intervention may have had a modest modulatory effect, but the influence was not sufficient to account for the major group differences observed. Nonetheless, the possibility of residual confounding cannot be fully excluded, and future studies with larger sample sizes, controlled probiotic administration, and longitudinal sampling are warranted to clarify the impact of probiotics on gut microbiota and ROP risk.

In conclusion, 16S rRNA sequencing and non-targeted metabolomics revealed characteristic changes in the intestinal flora and DAMs of ROP. ROP was usually diagnosed at week 4, and at this time, the α-diversity of intestinal flora in ROP was increased, with *Bifidobacterium*, *Rhodococcus*, *Staphyloococcus*, *Caulobacter*, *Sphingomonas*, *Aquabacterium*, and *Klebsiella* as the potential candidate biomarkers for ROP. In addition, the screened DAMs, including prostaglandin F3a and N-methyl-2-oxoglutaramate, and their enriched pathways of steroid hormone biosynthesis; PPAR signaling pathway; linoleic acid metabolism; histidine metabolism; and alanine, aspartate, and glutamate metabolism, may participate in the occurrence and progression of ROP. Our work identified crucial intestinal flora and key metabolites as underlying candidates in ROP, thus providing novel and promising targets or pathways for the clinical diagnosis and therapy of ROP.

## Data Availability

The raw metabolomics and 16S rRNA sequencing data from this study have been submitted to the OMIX database (accession number OMIX009126 for metabolomics) and NCBI (PRJNA1225445 for 16S rRNA). Additional data supporting the findings of this study can be obtained from the corresponding author upon request.

## Footnotes

1
https://github.com/SegataLab/lefse

2
https://github.com/picrust/picrust2/wiki

## Supplementary material

Supplementary Figure 1 Representative fundus photographs. **(A)** Representative fundus photograph of non-ROP infant. **(B)** Representative fundus photograph of ROP infant.

Supplementary Figure 2 Sub-analysis of 16S rRNA sequencing within the ROP group at T2. **(A)** Principal coordinate analysis (PCoA) based on the Bray–Curtis distance to compare the differences in community structure between the two groups at T2. **(B)** The α-diversity including ACE, Chao, Shannon, and Simpson indexes in the two different groups at the T2 time. **(C)** Top50 genera in the different groups at T2. **(D)** Bar plot showing the relative abundance of Bifidobacterium between the two groups.
