# Supplementary figures and images for: Multi-omics integration identifies key biomarkers in retinopathy of prematurity through 16S rRNA sequencing and metabolomics
Source: Front Microbiol. 2025 Jun 18;16:1601292. doi: 10.3389/fmicb.2025.1601292 (PMC12213487; doi:10.3389/fmicb.2025.1601292)

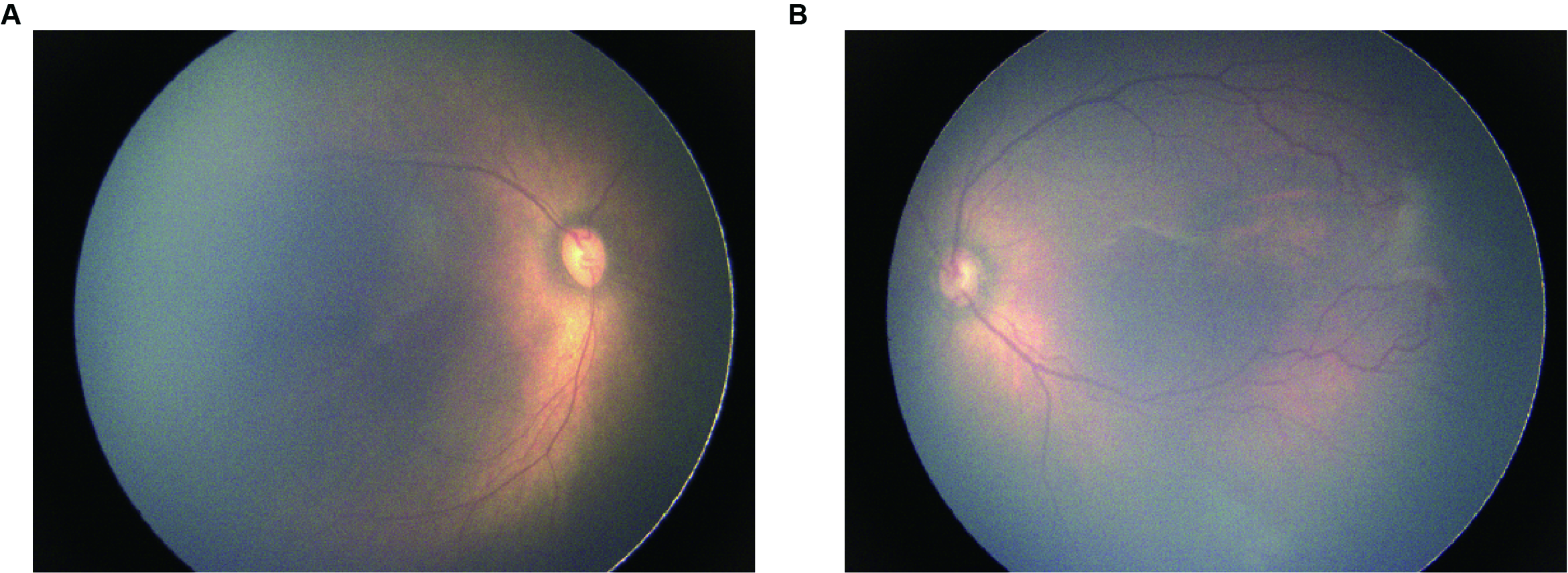

Supplement: Supplementary Figure 1 — Representative fundus photographs. (A) Representative fundus photograph of non-ROP infant. (B) Representative fundus photograph of ROP infant. [file Image_1.tif]

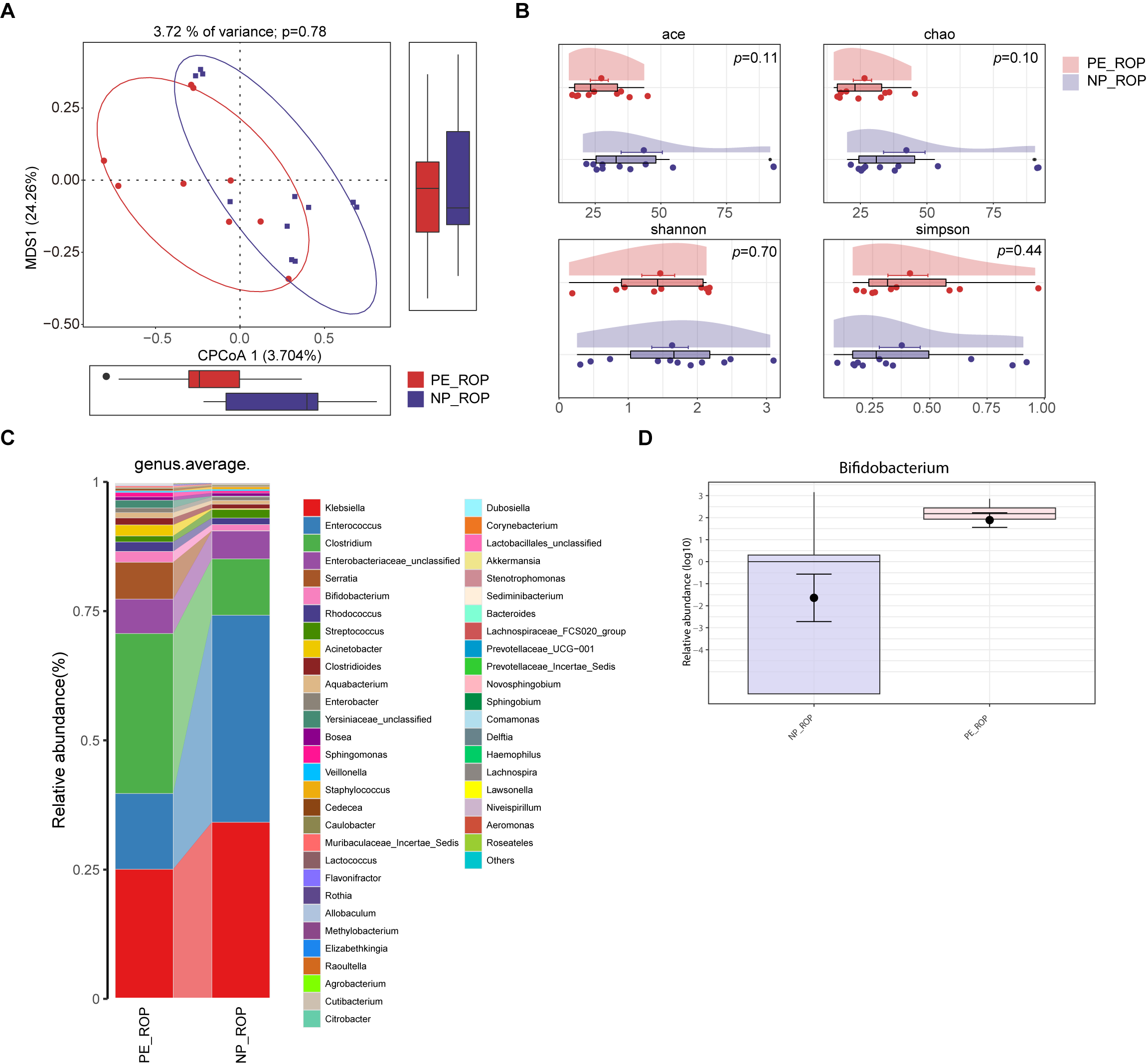

Supplement: Supplementary Figure 2 — Sub-analysis of 16S rRNA sequencing within the ROP group at T2. (A) Principal coordinate analysis (PCoA) based on the Bray–Curtis distance to compare the differences in community structure between the two groups at T2. (B) The α-diversity including ACE, Chao, Shannon, and Simpson indexes in the two different groups at the T2 time. (C) Top50 genera in the different groups at T2. (D) Bar plot showing the relative abundance of Bifidobacterium between the two groups. [file Image_2.tif]
